# Implementation and Effectiveness of the Power Over Pain Portal for Patients Awaiting a Tertiary Care Consultation for Chronic Pain: Protocol for a Pilot, Prospective, Cohort, Mixed Methods Study

**DOI:** 10.2196/64801

**Published:** 2025-09-25

**Authors:** Amin Zahrai, Etienne J Bisson, Yaadwinder Shergill, Danielle Rice, Natalie Zur Nedden, Lynn Cooper, Daniel James, Josh A Rash, Rachael Bosma, Tim Ramsay, Patricia Poulin

**Affiliations:** 1 Ottawa Hospital Research Institute Ottawa, ON Canada; 2 School of Epidemiology and Public Health University of Ottawa Ottawa, ON Canada; 3 Department of Anesthesiology and Perioperative Medicine Queen's University Kingston, ON Canada; 4 Chronic Pain Clinic Kingston Health Sciences Centre Kingston, ON Canada; 5 The Ottawa Hospital Pain Clinic The Ottawa Hospital Ottawa, ON Canada; 6 Department of Emergency Medicine University of Ottawa Ottawa, ON Canada; 7 Department of Anesthesiology and Pain Medicine University of Ottawa Ottawa, ON Canada; 8 Department of Psychology Memorial University of Newfoundland St-John's, NL Canada; 9 Centre for the Study of Pain University of Toronto Toronto, ON Canada; 10 Toronto Academic Pain Medicine Institute Women's College Hospital Toronto, ON Canada

**Keywords:** chronic pain, self-management, patient portal, virtual care, mental health, substance use, implementation science, feasibility study, protocol, online

## Abstract

**Background:**

Chronic pain (CP) affects approximately 8 million people in Canada. Access to CP care is challenging, and there is no robust monitoring system to support patient care and decision-making. The Power Over Pain (POP) Portal was developed by people living with CP, health care providers, researchers, health system decision-makers, policymakers, and community partners to address these concerns. The POP Portal is a comprehensive web-based platform that provides rapid access to a continuum of free, evidence-informed resources for the self-management of CP, mental health, and substance use health. The POP Portal also offers self-assessment tools that enable users to track their progress and receive personalized recommendations.

**Objective:**

This hybrid implementation-effectiveness type III pilot study aimed to determine the feasibility (ie, recruitment, integration, facilitators and barriers, patient engagement, usability, and acceptability) of the POP Portal’s implementation for people waiting for care at a tertiary pain clinic.

**Methods:**

A cohort of 80 adults living with pain was recruited from the waitlist of a tertiary care pain clinic over a 3-month period. Following an orientation on the POP Portal, participants were encouraged to use it according to their needs and preferences. They were also asked to complete questionnaires at baseline (0 months) and the 3-month follow-up. Primary feasibility measures included recruitment and retention rates and portal acceptability using the Acceptability E-scale. We also measured usability using the System Usability Scale, evaluated engagement through portal analytics, and identified facilitators and barriers via semistructured interviews with 12 to 15 study participants. These interviews further assessed the acceptability and usability of the portal for participants. Exploratory measures included pain severity, pain-related interference, self-efficacy, coping strategies, and symptoms of anxiety and depression.

**Results:**

We will present descriptive data on the cohort’s sex and gender, age, rural or urban status, and ethnic background, as well as the acceptability, usability, and feasibility of the portal. Measures of central tendency will be reported for continuous variables, and frequencies and proportions will be reported for categorical variables. We will also present change in clinical outcomes across time and a synthesis of qualitative and thematic data.

**Conclusions:**

We anticipate that most patients awaiting care at a tertiary pain clinic recruited will use the POP Portal and find it to be acceptable for addressing some of their pain and associated health concerns. If the feasibility of recruiting and retaining patients is demonstrated as anticipated, we will be able to move forward with a multisite study to evaluate the implementation and effectiveness of the POP Portal among patients waiting for a tertiary care consultation.

**International Registered Report Identifier (IRRID):**

DERR1-10.2196/64801

## Introduction

### Background

Chronic pain (CP) affects approximately 1 in 5 Canadians, including youth and adults, and costs the Canadian economy an estimated CAD $40 billion (US $28.9 billion) per year [1]. Approximately 60% of people with CP have co-occurring mental health disorders [2,3], and approximately 29% do not use opioids as prescribed [4]. Improving access to care for CP and co-occurring mental health and substance use concerns are among the field’s top research priorities [5]. People living with pain often wait for extended periods before receiving specialized pain care. While wait times in excess of 6 months have been deemed medically unacceptable [6], wait times for Canadian CP clinics have not changed over the last 10 years [7]. More than 50% of patients are not seen at these clinics within the recommended wait time, often waiting for several years [7], and as a result, they experience concomitant deterioration in function and quality of life [6].

Considering management options for CP, psychosocial interventions delivered virtually have been shown to be effective in improving pain interference, pain severity, psychological distress, and health-related quality of life when comparing to control groups [8]. Currently, no cohesive set of virtually delivered interventions for pain management exists within an integrated framework that augments the current continuum of care. Rather, there has been a reliance on stand-alone mobile apps, which present several issues that can impact their effectiveness and user adoption.

One concern is variability in the quality and reliability of the content provided, as not all apps are developed based on evidence-based practices [9]. In addition, there are privacy and data security concerns regarding storing information on multiple stand-alone apps as sensitive health information could be at risk if not properly protected [10]. The lack of personalized feedback and continuous outcome monitoring and the inability to adapt to individual patient needs can also limit the effectiveness of these apps [11]. Usability and accessibility are also significant concerns as older adults or those with limited technological proficiency may find these apps difficult to navigate [12]. Furthermore, while some are free, many apps require a purchase or subscription, which can create a financial barrier for some users, limiting equitable access to these potentially beneficial resources [13]. Finally, the engagement with and adherence to app-based interventions can be low as users might lose interest over time without proper motivation and support [12]. Addressing these issues is crucial for the successful integration of mobile apps into pain management strategies.

Our team (ie, a collaborative group of people living with pain, health care providers, researchers, health systems decision-makers, policymakers, intervention providers, and community partners) developed the Power Over Pain (POP) portal to remedy this gap. The POP Portal offers rapid access to free, evidence-informed virtual resources and interventions for the self-management of CP, mental health, and substance use aligned along a continuum of care. The portal also facilitates continuous outcome monitoring to provide feedback to people living with pain about their progress, promote behavior change, improve health decision-making, and enhance communication between health care providers and people living with pain. Importantly, the POP Portal was developed in accordance with the Stepped Care 2.0 model, a framework to integrate resources and interventions along a continuum of care that can support patients and their health care providers in achieving therapeutic goals. This model is resiliency based, grounded in recovery-oriented principles (eg, person driven and strength based), and self-corrective [14]. Stepped care approaches have been demonstrated to be acceptable and cost-effective for delivering mental health [15] and substance use [16] care. These approaches have also been studied in managing certain CP conditions such as low back pain [17], musculoskeletal pain [18], and osteoarthritis [19]. However, a 2019 review concluded that stepped care models for CP were inconsistently applied and that studies conducted on them were of low quality [20]. Our team adapted and implemented Stepped Care 2.0 for adult CP care at our tertiary care institution [21], which led to a substantial reduction in clinical appointment wait times [22]. The POP portal [23] was launched in November 2022 and reached 250,000 unique visitors in November 2024 through a promotional campaign targeting different relevant organizations in Canada. Many tertiary pain clinics in particular were enthusiastic to learn about the portal and its potential in improving pain care access. However, in addition to meeting discussions on how best to implement the portal in this context, strong evidence-based implementation strategies were lacking, limiting the success of portal use and benefits within tertiary pain clinics.

### Objectives

In preparation for an adequately powered multisite trial to evaluate the implementation and effectiveness of the POP Portal, this pilot study aimed to determine the feasibility of implementing the POP Portal for people living with pain who had been referred to a tertiary pain clinic in Canada and were awaiting their first appointment. The objectives of the pilot study were to determine the (1) feasibility of recruiting people living with pain who were waiting for their first appointment, (2) proportion of patients who agreed to have their health care number used for a future study (eg, impact of the POP Portal on health care use), (3) participant engagement with and usability and acceptability of the POP Portal, (4) facilitators of and barriers to the POP Portal’s implementation, (5) statistical parameters of effectiveness outcomes (outlined in the following sections), and (6) preliminary evidence of intervention effectiveness (ie, estimates of effect and variance for the secondary outcome).

## Methods

### Ethical Considerations

This protocol was reviewed and approved by the Ottawa Health Science Network Research Ethics Board, REB #20220443-01H. Participants were informed that their privacy would be respected and their personal information kept strictly confidential, unless release was required by law. Participants provided verbal informed consent before taking part in the study and were informed that they could withdraw their participation at any time and would not lose access to the portal if they chose to do so. The Ottawa Health Science Network Research Ethics Board may review relevant study records under the supervision of PP and their research staff for audit purposes. Adverse events that could reasonably be attributed to the use of the POP Portal were self-reported by the participants and tracked using an adverse event form. Study participants were not compensated for accessing the virtual POP Portal or its resources. Participation was incentivized in the following ways: participants who completed the 3-month follow-up questionnaire and interview were provided with a CAD $20 (US $14.46) and CAD $30 (US $21.69) Amazon gift card, respectively. Participants were not asked to pay fees for any part of this study.

### Study Design

**Figure 1:**
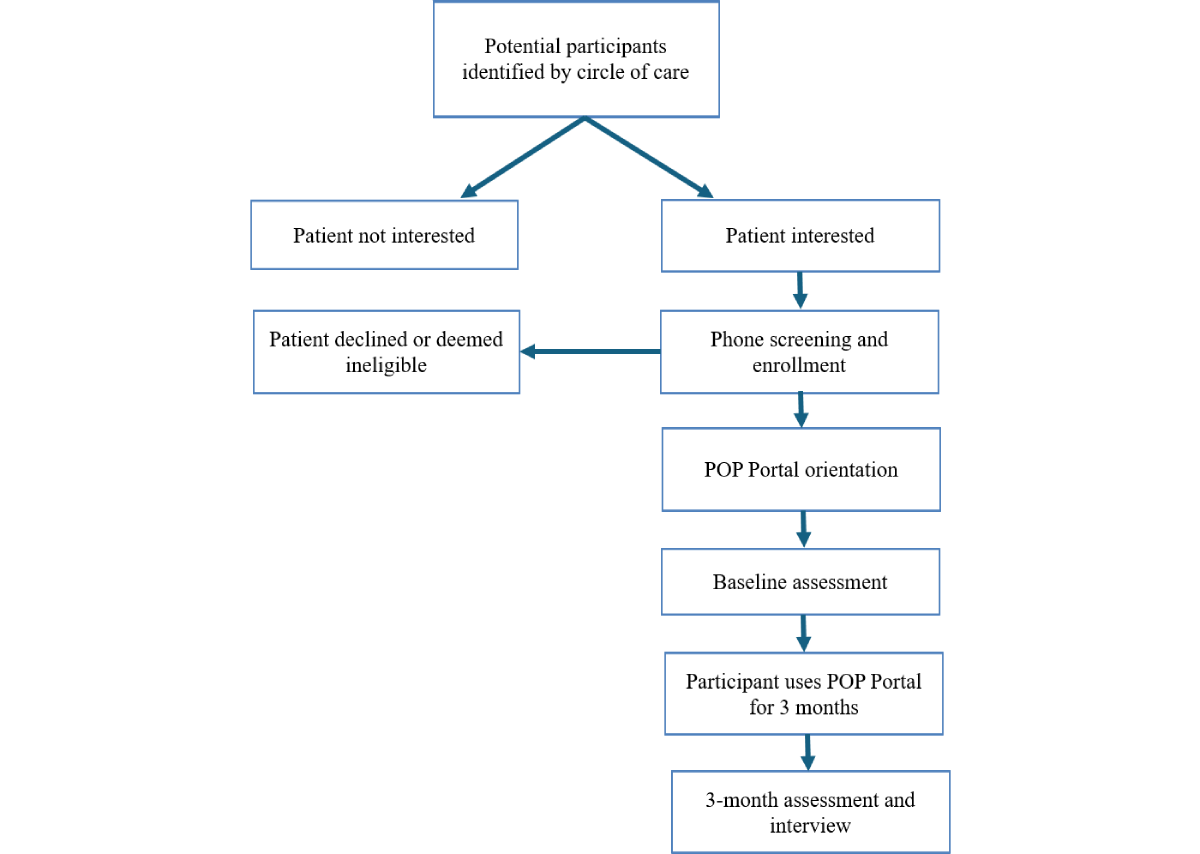
Workflow of the study. POP: Power Over Pain.

This project was a hybrid implementation-effectiveness type III pilot study using a prospective cohort mixed methods design. We used the SPIRIT (Standard Protocol Items: Recommendations for Interventional Trials) 2013 [24,25] checklist to guide our reporting for this protocol with adaptation for feasibility studies [25]. The enacted study will be reported in accordance with the CONSORT (Consolidated Standards of Reporting Trials) guidelines extension to randomized pilot and feasibility trials [26]. A workflow of the study can be found in Figure 1.

### Eligibility Criteria

Inclusion criteria were (1) adults (aged >18 years) referred to a tertiary care pain clinic (ie, awaiting triage or first appointment), (2) experience of CP (ie, ongoing, persistent, or recurrent pain for more than 3 months), (3) sufficient fluency in English or French to engage with the POP Portal’s programs and resources, (4) capacity to provide informed consent in English or French, (5) access to an electronic device with connection to the internet, and (6) agreement to be contacted for research.

Exclusion criteria were (1) self-reported barriers to the use of technology during the initial screening appointment, (2) experience of cancer-related pain, (3) inability to complete the study questionnaires and assessments, and (4) self-disclosure of an unmanaged mental health condition or suicidal ideations during the first study visit.

### Study Procedures

#### Overview

We identified prospective participants for this study in 2 ways. First, the pain clinic clerk called patients accepted for care at the pain clinic and informed them about the study; those who expressed an interest were referred to the study coordinator for screening and to discuss participation. Second, the study coordinator screened electronic health records to identify those whose consultation request had yet to be triaged and who had provided institutional permission to be contacted for research.

The study coordinator contacted eligible patients via phone. During this call, the coordinator described the study background, expectations of participation, voluntariness, rights to withdrawal, and risks and benefits and obtained verbal consent. The study coordinator ensured that verbal consent was recorded during this initial phone call. Consenting participants were sent a study information sheet along with a web link to complete a baseline questionnaire. In addition, the coordinator scheduled a videoconference session with participants to orient them to the POP Portal (see details in the following sections).

The study coordinator sent participants a web link to complete a follow-up assessment 3 months after the orientation session (Figure 1), and selected participants were invited to undergo follow-up interviews at this time (see the Data Collection and Outcomes section for more details).

#### Study Intervention Delivery

Following the initial call, participants took part in a 15-minute orientation session that introduced the POP Portal and its resources, including detailed information about (1) navigating the portal and its resources, (2) creating an account, (3) registering for courses, and (4) completing self-assessments. Following this session, participants were asked to use the POP Portal for a 3-month period. Participants could ask for guidance on any aspect of the portal covered in the orientation session at any time over this period (eg, usability difficulties). However, the participants were not provided with further instructions on what resources or services they should use on the POP Portal (ie, resource recommendations), nor were there restrictions on concomitant care or interventions.

#### Sample Size

We aimed to recruit 80 participants from a tertiary care pain clinic over a 3-month recruitment period. We anticipated recruiting 20 to 30 adults per month. For the qualitative interviews, we purposively (eg, sex, gender, pain type, duration with pain, and comfort with technology) invited 12 to 15 adults, which we anticipated would be sufficient to achieve saturation [27].

### Study Intervention

#### Overview

The POP Portal is a comprehensive virtual platform co-designed by a diverse group of health care providers, researchers, decision-makers, and Canadians who live with CP. The POP Portal (1) provides rapid access to free, evidence-informed virtual resources for CP, mental health, and substance use health arranged along a stepped care continuum of offerings; and (2) facilitates continuous outcome monitoring through optional self-assessments to allow for rapid adjustment to resource recommendations and program evaluation.

Recognizing the difference in the needs and preferences of youth and adult populations, 2 distinct portals were created to better serve each population. This study focused on an adult population, and thus, the adult POP Portal is described in the following sections.

#### Stepped Care Resources

In line with the Stepped Care 2.0 framework, the resources available on the POP Portal are designed to vary in intensity, corresponding to different levels of user commitment. These resources enhance a continuum of care and are accessible at any time, thereby enabling users to engage with them according to their individual preferences and needs.

##### Step 1: Educational Resources

Participants who wanted to learn basic facts about CP, including the relationship among CP, mental health, and substance use, had access to a range of evidence-informed educational resources (eg, articles, videos, and podcasts), including (1) Pain U Online, developed by the Toronto Academic Pain Medicine Institute, which covers topics on CP mechanisms; factors that influence pain; pharmacological, physical, and psychological strategies for pain management; insomnia; and a healthy lifestyle; and (2) LivePlanBe+, developed by Pain BC, which currently hosts 23 modules on pain science, nutrition, self-management techniques, sleep, symptom management, and communication, among others.

##### Step 2: Virtual Self-Guided CP Self-Management Courses

Participants were encouraged to create a free account to gain access to free virtual self-guided CP self-management courses, including (1) The Pain Course, developed at the eCentreClinic, Macquarie University; and 2) Empowered Management, developed by the Toronto Academic Pain Medicine Institute.

The Pain Course is an online 8-week cognitive behavioral therapy–based self-management program that includes six modules: (1) CP education, (2) understanding CP and the relationship between pain and emotions, (3) managing unhelpful thoughts, (4) managing uncertainty and problem-solving, (5) managing physical symptoms and maintaining physical activity while minimizing fatigue and pain exacerbation, and (6) maintenance strategies and relapse prevention. It has been evaluated among individuals with diverse CP conditions and shown to be acceptable and associated with improved pain, pain interference, depression, and anxiety [28-30].

Empowered Management is an online program specifically designed for patients living with CP who are waiting for tertiary pain care. It aims to improve their readiness for change and provide them with self-management skills that will enhance their interaction with health care providers in CP clinics. The modules include (1) setting expectations; (2) what is CP; (3) biopsychosocial factors and approaches to CP; (4) empowered management; (5) self-awareness, compassion, and acceptance; (6) values; (7) goal setting; and (8) communication. The program includes a reflection journal that accompanies the psychoeducational modules and weekly assignments [31]. It was recently tested in 2 Ontario Chronic Pain Network clinics and found to be acceptable and usable (personal communication, Rosemary Wilson, August 25, 2024).

##### Step 3: Peer Support

Participants had access to different peer support services to help them connect with peers, including peer support groups and health coaching.

Participants were able to connect with different communities of support (ie, peer support groups) and find a safe space to talk with people who may know better where they were coming from, including the People in Pain Network. The People in Pain Network is a national community of people with persistent pain helping other people with persistent pain improve the quality of their lives by offering education and support through monthly virtual and in-person meetings.

Participants had access to health coaching via Ontario Self-Management, a free one-on-one web-based support program to help people living with pain manage their condition. The health coaches are people with lived experience trained in self-management support and communication skills.

##### Step 4: Live Interactive Workshops

Participants had access to a suite of live interactive workshops covering various topics related to CP, mental health, and substance use health, some of which included pain neuroscience, sleep, nutrition, gentle movement, communication, pacing and planning, and engaging in meaningful activities.

##### Step 5: Individual Counseling

Participants who wished to access individual counseling were directed to free services available in their jurisdiction.

#### Self-Assessments

Regarding other POP Portal uses, participants had the option to complete self-assessments once every few weeks and were presented with a visual depiction of their results to help them track their symptoms over time. These self-assessments consisted of the following valid and reliable patient-reported scales: the Patient-Reported Outcomes Measurement Information System Pain Intensity instrument; Pain Disability Index; 2-item short form of the Pain Self-Efficacy Questionnaire; Patient Health Questionnaire–4; Patient-Reported Outcomes Measurement Information System Sleep Disturbance Short Form instrument; 1 item modified from the World Health Organization Alcohol, Smoking, and Substance Involvement Screening Test; and the Global Appraisal of Individual Needs substance use subscale. These scales are described in Table 1.

Once the participant completed a self-assessment, their results were displayed, and feedback was provided based on where they scored within predefined categorical groupings (eg, low, moderate, or high). Resource recommendations were preselected by our team for each domain measured (eg, pain-related disability and sleep disturbance) per scoring categorical groupings. The self-assessments were fully integrated within the POP Portal through an automated electronic data capture system maintained by our technology partner. The goal of the self-assessments was to provide personalized feedback generated by the platform about potential intervention targets for CP, mental health, or substance use health. We asked participants for consent to use their self-assessment data for this study.

**Table 1:** Description of the Power Over Pain Portal self-assessment measures.

Scale	Description
PROMIS^a^ Pain Intensity instrument	The PROMIS Pain Intensity instrument is a 1-item numerical rating scale from 0 to 10 for measuring pain intensity [32].
PDI^b^	The PDI is a 7-item form (including responsibility of home and family, self-care, occupation, sexual behavior, social activity, recreation, and life support activities) that assesses the magnitude of self-reported pain-related disability of the participant regardless of the pain area or pain-related diagnosis. The pain-related disability questions are scored on an 11-point numerical rating scale (0-10) [33].
PSEQ-2^c^	The PSEQ-2 is a 2-item survey with a 7-point numerical rating scale (0-6) to assess patients’ pain self-efficacy or belief in one’s capability to perform activities despite having pain. The PSEQ-2 has high validity and internal consistency, with evidence for its test-retest reliability, sensitivity to change, and convergent validity [34-36].
PHQ-4^d^	The PHQ-4 is a brief 4-item survey of core symptoms and signs of depression (PHQ-2) and anxiety (GAD-2). The total PHQ-4 score provides an overall measure of symptom burden as well as functional impairment and disability. Its total score (sum of 4 items) measures psychological distress rated as normal (0-2), mild (3-5), moderate (6-8), and severe (9-12); a GAD-2^e^ score of ≥3 suggests potential anxiety symptoms, and a PHQ-2^f^ score of ≥3 suggests potential depression symptoms [37].
PROMIS Sleep Disturbance Short Form	The PROMIS Sleep Disturbance Short Form is a 4-item form exploring the participants’ sleep characteristics and difficulties over the previous 7 days. The participant is asked to select, for the first question, from 1 of 5 options (“very poor,” “poor,” “fair,” “good,” or “very good”) and, for the last 3 questions, from 1 of the following 5 options for each question: “not at all,” “a little bit,” “somewhat,” “quite a bit,” and “very much” [38].
WHO-ASSIST^g^	A single item modified from the WHO-ASSIST will be used to evaluate the risk associated with the client’s substance use and whether this use is hazardous and likely to cause harm (now or in the future) if it continues. We ask patients to indicate whether they have concerns regarding any of the substances from a list provided to them [39].
GAIN^h^ Short Screener	The GAIN Short Screener will be used to identify individuals who are experiencing challenges or are at risk of developing challenges as it pertains to mental and substance use issues. We administer 5 items to the participants, each scored on a 4-point numerical rating scale (0-3) [40,41].

^a^PROMIS: Patient-Reported Outcomes Measurement Information System.

^b^PDI: Pain Disability Index.

^c^PSEQ-2: 2-item short form of the Pain Self-Efficacy Questionnaire.

^d^PHQ-4: Patient Health Questionnaire–4.

^e^GAD-2: 2-item Generalized Anxiety Disorder scale.

^f^PHQ-2: Patient Health Questionnaire–2.

^g^WHO-ASSIST: World Health Organization Alcohol, Smoking, and Substance Involvement Screening Test.

^h^GAIN: Global Appraisal of Individual Needs.

### Data Collection and Outcomes

#### Overview

Outcomes for this pilot study were derived through advisory committee meetings with patient partners who are people living with pain, previous research in the field, and clinical experience of the multidisciplinary team at the tertiary care pain clinic (eg, clinicians, nurses, psychologists, and social workers). Participants were asked to complete a baseline assessment and a 3-month assessment (after portal use) through a web-based survey (LimeSurvey GmbH). Scales included as primary and secondary feasibility outcomes were collected only at the 3-month assessment, whereas all exploratory clinical effectiveness outcomes were collected at both baseline and the 3-month assessment.

#### Primary Feasibility Outcomes and Interpretation

Our primary feasibility outcomes were recruitment rate (ie, number of patients consenting), 3-month retention rate (ie, 3-month assessment completion), and participant acceptability assessed using the Acceptability E-scale 3 months after participant recruitment [42] and through interviews. The Acceptability E-scale is a 6-item questionnaire that uses a 5-point Likert scale to evaluate participants’ experiences with the program. The form has been shown to have strong psychometric properties to assess participants’ acceptance and perception of digital health interventions [42,43].

#### Secondary Feasibility Outcomes

Secondary feasibility outcomes comprised (1) rates of participant accrual, dropout, screening, eligibility, and 3-month assessment completion; (2) participants’ satisfaction, assessed using the Acceptability E-scale 3 months after participant recruitment [42]; (3) participants’ perceived barriers and facilitators regarding the portal, which will be tracked throughout the study and through participant interviews (see the following section); and (4) portal usability, assessed by the participants using the System Usability Scale 3 months after recruitment [44]. The System Usability Scale is a 10-item questionnaire asking participants about the usability of the POP Portal. Each question has a 5-point numerical rating scale (1-5), with 1 indicating strong disagreement and 5 indicating strong agreement. It has been shown to be both valid and reliable in providing a global view of subjective assessments of usability [44-46].

We also explored participant engagement with the portal and its resources throughout the 3 months of portal use through system analytics. We collected metrics that were maintained by our technology partner and guided by those that were collected for Wellness Together Canada [47], including sign-ups; sign-ins; first-time user activation (ie, rate at which new users engage in a meaningful way with the portal, including assessment completion, or view of progress over time); type, nature, and frequency of resources accessed; number of self-assessments completed; participant retention (eg, 1 week vs 3 months after study enrollment); and impact (eg, correlation between participants’ improvement in pain, mood, or substance use and resource use).

#### Interviews

We conducted semistructured interviews with a random sample of 12 to 15 participants who used the POP Portal’s resources or courses to further assess the acceptability, usability, and impact of the POP Portal. Interviews spanned 30 to 45 minutes in duration and were conducted via Microsoft Teams. We developed an interview guide using components of the theoretical domains framework [48], theoretical framework of acceptability [49], the System Usability Scale [44], and theoretical domains framework questionnaire in implementation research [50]. The interview guide was refined through discussion with pain experts across Canada and with people living with pain. The guide was intended to provide structure and context for the participants’ responses. Through these interviews, we aimed to better understand the experience of participants with the resources offered to them in the POP Portal, what they liked or disliked about it, benefits and harms experienced from the portal, level of confidence in and comfort with the portal, barriers to the portal’s implementation or delivery, and potential improvements or additions. Interviews were conducted by trained study research staff, recorded, and transcribed verbatim.

#### Exploratory Clinical Effectiveness Outcomes

Informed by a consensus-driven minimum dataset [51] for adults with CP from the Centre hospitalier de l’Université de Montréal (University of Montreal Health Centre), participants’ pain type, location, onset, duration, frequency, and diagnosis were collected if known. We also examined whether there were changes in the following clinical outcomes from before enrollment to 3 months after enrollment: (1) pain intensity and interference (Brief Pain Inventory), (2) pain self-efficacy (2-item short form of the Pain Self-Efficacy Questionnaire), (3) pain coping skills (Chronic Pain Coping Inventory), (4) attitudes toward and beliefs about CP (Survey of Pain Attitudes), (5) health-related quality of life (12-Item Short Form Health Survey version 2), (6) symptoms of anxiety (7-item Generalized Anxiety Disorder scale), and (7) symptoms of depression (Patient Health Questionnaire–8). We also assessed participants’ perceived overall effectiveness using the Patient Global Impression of Change at the follow-up visit. These scales are described in Table 2.

**Table 2:** Description of study exploratory clinical effectiveness outcomes.

Scale	Description
BPI^a^	The BPI will be used to measure pain severity (4 items on an 11-point numerical rating scale) and pain interference with daily functioning (7 items on an 11-point numerical rating scale). Participants’ “worst pain” or the arithmetic mean of the 4 severity items can be used as a measure of pain severity; the arithmetic mean of the 7 interference items (general activity, walking, work, mood, enjoyment of life, relations with others, and sleep) can be used as a measure of pain interference. The BPI has sufficient reliability (Cronbach α coefficients are frequently higher than 0.80), construct validity, and responsiveness in several pain and other populations [52-57]. Worse pain and average pain scores of 1-4 are viewed as indicative of mild pain, scores of 5-6 are viewed as indicative of moderate pain, and scores of 7-10 are viewed as indicative of severe pain [58].
PSEQ-2^b^	The PSEQ-2 will be used similarly to its use for the portal’s self-assessments described in Table 1.
CPCI^c^	The CPCI is an 8-item questionnaire covering the use of pain coping skills, including the scales on guarding, resting, asking for assistance, relaxation, task persistence, exercise and stretch, seeking, and coping self-statements. It asks the patient to indicate the number of days during the previous week in which they used each of the strategies to deal with pain. The questionnaire has been shown to have strong internal consistency reliability, test-retest stability, and validity in CP^d^ populations [59,60].
SOPA^e^	The SOPA is a 7-item form to assess patients’ attitudes toward and beliefs about their CP. It includes scales on pain control, disability, harm, emotion, medication, solicitude, and medical cure. Patients are asked to indicate how much they agree or disagree with each statement on a 5-point numerical rating scale (0-4). The survey has good internal consistency, test-retest reliability, and convergent and discriminant validity [61].
SF-12^f^ version 2	The SF-12 version 2 is a 12-item survey that measures health-related quality of life, functional health, and well-being across physical and mental health domains. We will evaluate limitations to participants’ physical activities due to their current health using the physical functioning subscale. The SF-12 version 2 has shown good psychometric validity and reliability for evaluating health-related quality of life in both general [62-65] and specific populations, including those with CP [66], cancer [67], hemophilia [68], mental illnesses, and behavioral health diagnoses [69], among others.
GAD-7^g^	The GAD-7 is a 7-item scale used to screen for potential signs and symptoms of anxiety and assess the severity of generalized anxiety disorder. Scores range from 0 to 21, and a clinically meaningful change is 5 points or more. The GAD-7 has high internal consistency and convergent validity (Cronbach α values are frequently above 0.82) across heterogeneous psychiatric populations [70,71], as well as sound diagnostic validity, with sensitivity of 0.66 to 0.89 and specificity of 0.80 to 0.82 for generalized anxiety disorder and anxiety disorders including social anxiety, posttraumatic stress disorder, and panic disorder [72,73].
PHQ-8^h^	The PHQ-8 is an 8-item scale to assess the frequency of depressive symptoms, with scores ranging from 0 to 24. A total score of 0-4 represents no significant depressive symptoms, a score of 5-9 represents mild depressive symptoms, a score of 10-14 represents moderate depressive symptoms, a score of 15-19 represents moderately severe depressive symptoms, and a score of 20-24 represents severe depressive symptoms. The PHQ-8 has been shown to be reliable and have good construct and criterion validity to screen for depression in patients with heart failure and in the general population [74-76].
PGI-C^i^	The PGI-C scale [77] is a 1-item scale to evaluate the perceived effect of disease management, asking the participants about their overall status at follow-up; it includes 7 options, ranging from “very much worse” to “very much improved.” The scale has demonstrated to have high test-retest reliability and is a potentially clinically meaningful measure for a variety of pain populations, with evidence of good validity [77-80].

^a^BPI: Brief Pain Inventory.

^b^PSEQ-2: 2-item short form of the Pain Self-Efficacy Questionnaire.

^c^CPCI: Chronic Pain Coping Inventory.

^d^CP: chronic pain.

^e^SOPA: Survey of Pain Attitudes.

^f^SF-12: 12-Item Short Form Health Survey.

^g^GAD-7: 7-item Generalized Anxiety Disorder scale.

^h^PHQ-8: Patient Health Questionnaire–8.

^i^PGI-C: Patient Global Impression of Change.

#### Demographic Characteristics

We collected age, sex and gender, ethnic background, and the first 3 digits of participants’ postal codes from medical records to provide basic demographic characteristics.

### Data Management

Personal information was kept confidential unless release was required by law. Participants were identified in study data through a unique study identification number. Project data were encrypted, password protected, and locally stored on The Ottawa Hospital’s Microsoft 365 SharePoint or OneDrive subject to the institution’s policies and procedures regarding security and backup. Questionnaire data were collected using LimeSurvey and then downloaded by the study coordinator at least biweekly into a spreadsheet securely stored on the institution’s Microsoft 365 SharePoint or OneDrive. Data were deleted from LimeSurvey after all study data were securely transferred and stored locally on the research institution’s network. Only the research team members directly involved in conducting the research had access to the data. Passwords were stored in a master list, with only the principal investigator, research program manager, and study coordinator having access.

The data safety and monitoring committee was guided by a charter of roles and responsibilities and consisted of a statistical expert, a person with lived experience of CP, a pain medicine specialist, and a health psychologist who are independent of the research team. This group met with the study steering committee on a monthly basis during study recruitment and intervention to review recruitment, accumulating study data, and adverse events and provided guidance regarding any needed action.

### Data Analysis

#### Quantitative Analyses

The primary feasibility outcomes and secondary outcomes previously mentioned were described using descriptive statistics (frequencies and proportions), point estimates, and 95% CIs. For this pilot study, we did not stratify or analyze the acceptability or usability outcomes by type or amount of resources used. Even though we were underpowered for efficacy, we explored ranges of effect sizes using point estimates of change across time and associated 95% CIs for each resource [81]. Graphs and tables of descriptive data were prepared. Study data were imported into SPSS (IBM Corp) for statistical analysis. Open-text responses were reviewed by the study team for converging themes. Missing data were handled using multiple imputation; single-point estimates were determined using parameter estimates and SEs of 10 imputed datasets.

#### Qualitative Analyses

Interviews were transcribed, and analyses and coding were completed by 2 research staff members using the NVivo software (QSR International) and following the method described by Saldaña [82]. The study staff acquainted themselves with the data through reading the transcripts independently and started developing the codebook. They then met to compare coding, with any disagreements resolved through consensus or discussion with the senior author. We then used a deductive thematic analysis approach [48] to map emerging categories and themes to constructs within the theoretical framework of acceptability and theoretical domains framework. We triangulated our data by comparing themes identified during the analyses of participant interviews with quantitative data collected to validate our findings and expand our understanding of the effectiveness, acceptability, and usability of the portal wherever possible

### Feasibility Interpretation

Following recommendations for feasibility studies, we developed a priori criteria on our primary feasibility outcomes to indicate whether progression to an adequately powered trial was feasible. The a priori criteria were organized through a traffic light system (Textbox 1).

**A priori feasibility criteria.**

- Green: continue without modifications; this will be indicated if (1) we recruit a minimum of 80 adults at the tertiary care pain clinic over 3 months, (2) we achieve a minimum of 80% retention rate (ie, participants completing the Pain Course resource delivered through the Power Over Pain [POP] Portal and pretest-posttest outcome measures), and (3) most (≥70%) of the participants deem the POP Portal to be acceptable for addressing some of their pain and associated health concerns, as measured using study questionnaires, portal self-assessments, or interviews.
- Yellow: continue but modify protocol with close monitoring; this will be indicated if we recruit 40-79 adult participants over 3 months, achieve a 50%-79% retention rate, or 50%-69% of the participants find the POP Portal acceptable.
- Red: definitive trial not feasible; this will be indicated if we recruit <40 adult participants over 3 months, achieve a <50% retention rate, or <50% of the participants find the POP Portal acceptable.

## Results

The Ottawa Health Science Network Research Ethics Board cleared all study procedures and materials for ethical compliance on October 24, 2022, in their initial version. The protocol in the current and final version (version 2) was cleared on May 16, 2023. Participants were able to enroll in the study between March 25, 2023, and August 7, 2023. Data collection was extended to November 18, 2023. Following the International Committee of Medical Journal Editors guidelines for authorship, the results were published in an open access journal—*Digital Health*; a qualitative manuscript was published in October 2024, and a quantitative manuscript was published in March 2025. Findings were also disseminated to different knowledge users of the portal through presentations and webinars offered by POP partners. No protocol deviations were noted.

## Discussion

### Expected Findings

Most patients referred to tertiary care for pain management have not had access to pain education, self-management, and peer support before referral. The POP Portal fills that gap by providing rapid access to a stepped care continuum of virtual self-management resources for CP. This study tested the feasibility and explored the effects of implementing the POP Portal in tertiary care with the hope of conducting a fully powered multisite implementation-effectiveness trial. As patients await their first visit to a tertiary care pain program (which could take months), we anticipate that (1) 80% of approached patients will be interested in being informed about the portal, (2) 70% of patients oriented to the portal will access some of its resources, and (3) most (≥70%) of the participants who use the portal will report it to be acceptable for addressing some of their pain and associated health concerns. In terms of feasibility of the study protocol, we anticipate the ability to recruit a minimum of 80 participants over 3 months and achieve a minimum of 80% retention rate. If our anticipated findings are true, we will be able to progress to an adequately powered multisite trial without modifications to the protocol.

Studies looking at the implementation of a comprehensive portal providing evidence-informed online resources on education, self-management, and peer support to empower patients with a health condition are limited. During the COVID-19 pandemic, Wellness Together Canada was quickly developed and successfully implemented, providing much needed access to online mental health resources [83]. While the platform is no longer active, Wellness Together Canada was one of the first examples of the benefits of providing a range of resources and services on a stepped care continuum at a population level and was the inspiration for the development of the POP Portal. As previously mentioned, our team developed 2 distinct portals, one for adults and one for youth living with CP. A hybrid implementation-effectiveness trial is currently underway, testing the Youth Portal (popyouth.ca) across Canada [84].

There are a multitude of interventions for self-management of pain that have been developed [85]. For example, The Pain Course was developed and well tested, showing efficacy across different populations [28-30]. However, most interventions being developed rarely become available or accessible to the public [1]. A platform such as the POP Portal provides a space to host these resources and make them accessible to the public (outside the research sphere). It also provides the opportunity to continue the evaluation of those resources at a larger scale using a real-world pragmatic approach.

### Limitations

The POP Portal is an online platform that provides virtual resources. We acknowledge that people with no internet access or low digital literacy will not derive equal benefit from this type of innovation. Although no explicit strategies were in place at the time of study start to reach these groups, we planned to document reasons to decline participation in the feasibility study to inform strategies in future studies. This study also used a simple pretest-posttest design for outcome measures, which could limit the capture of user experience evolution. Multiple time points during the intervention should be considered for future similar protocols. As a first step in the implementation of the POP Portal, this study relied primarily on self-reported engagement with the portal (eg, interviews and scales) rather than system analytics, which could be seen as a limitation in terms of fidelity of portal use. However, we have observed that the quality and usefulness of engagement, even if they are minimal (eg, use of a single resource), can translate into meaningful improvement. This type of information is better captured through interviews.

### Future Directions

Our ultimate goal with the POP Portal is to improve care access for people living with pain across Canada so that they can receive the appropriate care at the appropriate time according to their needs and preferences. To reach this goal, our future directions are 3-fold. First, regarding portal improvement, implementation, and evaluation, we will continue the development of the POP Portal based on studies evaluating the experiences of users and gathering feedback on portal improvement (eg, interviews, focus groups, and mass surveying). Using implementation science frameworks, we will conduct studies examining barriers to and facilitators of the implementation of the POP Portal in different contexts, such as tertiary care and primary care [86] as well as remote rural and Indigenous communities [87]. Finally, we will also conduct studies to evaluate the effectiveness of the portal in its entirety but also of specific components of it (eg, the effects of taking a self-directed course on the portal). We will also assess how users navigate the portal using system analytics of the POP Portal platform.

### Conclusions

The POP Portal aims to empower people living with CP and associated mental health or substance use health needs with rapid access to flexible, responsive, and individualized resources to improve their overall quality of life and functioning. We anticipated that most recruited patients awaiting care at a tertiary pain clinic will use the POP Portal and find it to be acceptable for addressing some of their pain and associated health concerns. If the feasibility of recruiting and retaining patients is demonstrated as anticipated, we will move forward with a definitive multisite study evaluating the implementation and effectiveness of the POP Portal among patients waiting for a tertiary care consultation.

## Abbreviations

CONSORT: Consolidated Standards of Reporting Trials
CP: chronic pain
POP: Power Over Pain
SPIRIT: Standard Protocol Items: Recommendations for Interventional Trials

## References

[1] Chronic pain in Canada: laying a foundation for action. Canadian Pain Task Force, Health Canada.

[2] Rayner L, Hotopf M, Petkova H, Matcham F, Simpson A, McCracken LM (2016). Depression in patients with chronic pain attending a specialised pain treatment centre: prevalence and impact on health care costs. Pain.

[3] Sagheer MA, Khan MF, Sharif S (2013). Association between chronic low back pain, anxiety and depression in patients at a tertiary care centre. J Pak Med Assoc.

[4] Tetsunaga T, Tetsunaga T, Nishida K, Kanzaki H, Misawa H, Takigawa T, Shiozaki Y, Ozaki T (2018). Drug dependence in patients with chronic pain: a retrospective study. Medicine (Baltimore).

[5] Poulin P, Shergill Y, Romanow H, Busse JW, Chambers CT, Cooper L, Forgeron PA, Olsen Harper A, Hudspith M, Iorio A, Lalloo C, Ouellette C, Robertson R, Smeenk S, Stevens B, Stinson J (2018). Researching what matters to improve chronic pain care in Canada: a priority-setting partnership process to support patient-oriented research. Can J Pain.

[6] Lynch ME, Campbell FA, Clark AJ, Dunbar MJ, Goldstein D, Peng P, Stinson J, Tupper H, Canadian Pain Society Wait Times Task Force (2007). Waiting for treatment for chronic pain - a survey of existing benchmarks: toward establishing evidence-based benchmarks for medically acceptable waiting times. Pain Res Manag.

[7] Choinière M, Peng P, Gilron I, Buckley N, Williamson O, Janelle-Montcalm A, Baerg K, Boulanger A, Di Renna T, Finley GA, Intrater H, Lau B, Pereira J (2020). Accessing care in multidisciplinary pain treatment facilities continues to be a challenge in Canada. Reg Anesth Pain Med.

[8] Slattery BW, Haugh S, O'Connor L, Francis K, Dwyer CP, O'Higgins S, Egan J, McGuire BE (2019). An evaluation of the effectiveness of the modalities used to deliver electronic health interventions for chronic pain: systematic review with network meta-analysis. J Med Internet Res.

[9] Rosser BA, Eccleston C (2011). Smartphone applications for pain management. J Telemed Telecare.

[10] Madden M, Gilman M, Levy K (2017). Privacy, poverty, and big data: a matrix of vulnerabilities for poor Americans. Wash U L Rev.

[11] Kumar S, Nilsen WJ, Abernethy A, Atienza A, Patrick K, Pavel M, Riley WT, Shar A, Spring B, Spruijt-Metz D, Hedeker D, Honavar V, Kravitz R, Lefebvre RC, Mohr DC, Murphy SA, Quinn C, Shusterman V, Swendeman D (2013). Mobile health technology evaluation: the mHealth evidence workshop. Am J Prev Med.

[12] Birkhoff SD, Smeltzer SC (2017). Perceptions of smartphone user-centered mobile health tracking apps across various chronic illness populations: an integrative review. J Nurs Scholarsh.

[13] Elbert NJ, van Os-Medendorp H, van Renselaar W, Ekeland AG, Hakkaart-van Roijen L, Raat H, Nijsten TE, Pasmans SG (2014). Effectiveness and cost-effectiveness of ehealth interventions in somatic diseases: a systematic review of systematic reviews and meta-analyses. J Med Internet Res.

[14] Newfoundland and Labrador stepped care 2.0 e-mental health demonstration project. Mental Health Commission of Canada.

[15] Ho FY, Yeung W, Ng TH, Chan CS (2016). The efficacy and cost-effectiveness of stepped care prevention and treatment for depressive and/or anxiety disorders: a systematic review and meta-analysis. Sci Rep.

[16] Babor TF, McRee BG, Kassebaum PA, Grimaldi PL, Ahmed K, Bray J (2007). Screening, Brief Intervention, and Referral to Treatment (SBIRT): toward a public health approach to the management of substance abuse. Subst Abuse.

[17] Von Korff M, Moore JC (2001). Stepped care for back pain: activating approaches for primary care. Ann Intern Med.

[18] Comer C, Glover J, Richardson J, Yaseen R, Foster S, Wolfenden NM, Hughes GJ (2016). Stratification of treatment in a community-based musculoskeletal service: a mixed-methods study to assess predictors of requiring complex care. Arch Phys Med Rehabil.

[19] Smink AJ, van den Ende CH, Vliet Vlieland TP, Bijlsma JW, Swierstra BA, Kortland JH, Voorn TB, Teerenstra S, Schers HJ, Dekker J, Bierma-Zeinstra SM (2014). Effect of stepped care on health outcomes in patients with osteoarthritis: an observational study in Dutch general practice. Br J Gen Pract.

[20] Palylyk-Colwell E, Wright M (2019). Tiered care for chronic non-malignant pain: a review of clinical effectiveness, cost-effectiveness, and guidelines. Canadian Agency for Drugs and Technologies in Health.

[21] Bell L, Cornish P, Gauthier R, Kargus C, Rash J, Robbins R, Ward S, Poulin PA (2020). Implementation of the Ottawa Hospital Pain Clinic stepped care program: a preliminary report. Can J Pain.

[22] Lynch ME, Williamson OD, Banfield JC (2020). COVID-19 impact and response by Canadian pain clinics: a national survey of adult pain clinics. Can J Pain.

[23] Power Over Pain Portal.

[24] Chan AW, Tetzlaff JM, Altman DG, Laupacis A, Gøtzsche PC, Krleža-Jerić K, Hróbjartsson A, Mann H, Dickersin K, Berlin JA, Doré CJ, Parulekar WR, Summerskill WS, Groves T, Schulz KF, Sox HC, Rockhold FW, Rennie D, Moher D (2013). SPIRIT 2013 statement: defining standard protocol items for clinical trials. Ann Intern Med.

[25] Thabane L, Lancaster G (2019). A guide to the reporting of protocols of pilot and feasibility trials. Pilot Feasibility Stud.

[26] Eldridge SM, Chan CL, Campbell MJ, Bond CM, Hopewell S, Thabane L, Lancaster GA, PAFS consensus group (2016). CONSORT 2010 statement: extension to randomised pilot and feasibility trials. Pilot Feasibility Stud.

[27] Baker SE, Edwards R (2012). How many qualitative interviews is enough?. National Centre for Research Methods.

[28] Dear BF, Titov N, Perry KN, Johnston L, Wootton BM, Terides MD, Rapee RM, Hudson JL (2013). The Pain Course: a randomised controlled trial of a clinician-guided Internet-delivered cognitive behaviour therapy program for managing chronic pain and emotional well-being. Pain.

[29] Dear BF, Gandy M, Karin E, Staples LG, Johnston L, Fogliati VJ, Wootton BM, Terides MD, Kayrouz R, Perry KN, Sharpe L, Nicholas MK, Titov N (2015). The Pain Course: a randomised controlled trial examining an internet-delivered pain management program when provided with different levels of clinician support. Pain.

[30] Hadjistavropoulos HD, Schneider LH, Hadjistavropoulos T, Titov N, Dear BF (2018). Effectiveness, acceptability and feasibility of an internet-delivered cognitive behavioral pain management program in a routine online therapy clinic in Canada. Can J Pain.

[31] Bosma R, Bisson EJ, Cooper LK, Salomons TV, Galica J, Wilson R (2023). Experience-based design: empowering individuals while they wait for interprofessional chronic pain care. Patient Educ Couns.

[32] Askew RL, Cook KF, Revicki DA, Cella D, Amtmann D (2016). Evidence from diverse clinical populations supported clinical validity of PROMIS pain interference and pain behavior. J Clin Epidemiol.

[33] Giordano PC, Alexandre NM, Rodrigues RC, Coluci MZ (2012). The Pain Disability Questionnaire: a reliability and validity study. Rev Lat Am Enfermagem.

[34] Dubé MO, Langevin P, Roy JS (2021). Measurement properties of the Pain Self-Efficacy Questionnaire in populations with musculoskeletal disorders: a systematic review. Pain Rep.

[35] Nicholas MK, McGuire BE, Asghari A (2015). A 2-item short form of the Pain Self-efficacy Questionnaire: development and psychometric evaluation of PSEQ-2. J Pain.

[36] Adachi T, Enomoto K, Yamada K, Inoue D, Nakanishi M, Takahashi N, Nishigami T, Shibata M (2019). Evaluating the psychometric properties of two-item and four-item short forms of the Japanese Pain Self-Efficacy Questionnaire: a cross-sectional study. J Anesth.

[37] Löwe B, Wahl I, Rose M, Spitzer C, Glaesmer H, Wingenfeld K, Schneider A, Brähler E (2010). A 4-item measure of depression and anxiety: validation and standardization of the Patient Health Questionnaire-4 (PHQ-4) in the general population. J Affect Disord.

[38] Yu L, Buysse DJ, Germain A, Moul DE, Stover A, Dodds NE, Johnston KL, Pilkonis PA (2011). Development of short forms from the PROMIS™ sleep disturbance and Sleep-Related Impairment item banks. Behav Sleep Med.

[39] WHO ASSIST Working Group (2002). The Alcohol, Smoking and Substance Involvement Screening Test (ASSIST): development, reliability and feasibility. Addiction.

[40] Dennis ML, Feeney T, Stevens LH GAIN-SS Global Appraisal of Individual Needs-Short Screener (GAIN-SS): administration and scoring manual version 2.0.1. Chestnut Health Systems.

[41] Dennis ML, Chan YF, Funk RR (2006). Development and validation of the GAIN Short Screener (GSS) for internalizing, externalizing and substance use disorders and crime/violence problems among adolescents and adults. Am J Addict.

[42] Tariman JD, Berry DL, Halpenny B, Wolpin S, Schepp K (2011). Validation and testing of the Acceptability E-scale for web-based patient-reported outcomes in cancer care. Appl Nurs Res.

[43] Underhill ML, Hong F, Jones T, Sprunck-Harrild K, Walsh SK, Boyajian R, Berry DL, Partridge A (2017). Feasibility and acceptability of a web site to promote survivorship care in survivors of Hodgkin disease. JCO Clin Cancer Inform.

[44] Brooke J, Jordan PW, Thomas B, McClelland IL, Weerdmeester B (1996). SUS - a quick and dirty usability scale Usability and context. Usability Evaluation in Industry.

[45] Peres SC, Pham T, Phillips R (2013). Validation of the System Usability Scale (SUS). Proc Hum Factors Ergon Soc Annu Meet.

[46] Sauro J (2011). A Practical Guide to the System Usability Scale: Background, Benchmarks & Best Practices.

[47] Wellness Together Canada – support is just a click or call away. Government of Canada.

[48] Boyatzis R (1998). Transforming Qualitative Information: Thematic Analysis and Code Development.

[49] Sekhon M, Cartwright M, Francis JJ (2022). Development of a theory-informed questionnaire to assess the acceptability of healthcare interventions. BMC Health Serv Res.

[50] Huijg JM, Gebhardt WA, Crone MR, Dusseldorp E, Presseau J (2014). Discriminant content validity of a theoretical domains framework questionnaire for use in implementation research. Implement Sci.

[51] Lacasse A, Roy JS, Parent AJ, Noushi N, Odenigbo C, Pagé G, Beaudet N, Choinière M, Stone LS, Ware MA, Quebec Pain Research Network's Steering Committee of the Low Back Pain Strategic Initiative (2017). The Canadian minimum dataset for chronic low back pain research: a cross-cultural adaptation of the National Institutes of Health Task Force Research Standards. CMAJ Open.

[52] Jumbo SU, MacDermid JC, Kalu ME, Packham TL, Athwal GS, Faber KJ (2021). Measurement Properties of the Brief Pain Inventory-Short Form (BPI-SF) and Revised Short McGill Pain Questionnaire Version-2 (SF-MPQ-2) in pain-related musculoskeletal conditions: a systematic review. Clin J Pain.

[53] Keller S, Bann CM, Dodd SL, Schein J, Mendoza TR, Cleeland CS (2004). Validity of the brief pain inventory for use in documenting the outcomes of patients with noncancer pain. Clin J Pain.

[54] Tan G, Jensen MP, Thornby JI, Shanti BF (2004). Validation of the Brief Pain Inventory for chronic nonmalignant pain. J Pain.

[55] Gjeilo KH, Stenseth R, Wahba A, Lydersen S, Klepstad P (2007). Validation of the brief pain inventory in patients six months after cardiac surgery. J Pain Symptom Manage.

[56] Jelsness-Jørgensen LP, Moum B, Grimstad T, Jahnsen J, Opheim R, Prytz Berset I, Hovde ?, Torp R, Frigstad SO, Huppertz-Hauss G, Bernklev T (2016). Validity, reliability, and responsiveness of the brief pain inventory in inflammatory bowel disease. Can J Gastroenterol Hepatol.

[57] Kapstad H, Rokne B, Stavem K (2010). Psychometric properties of the brief pain inventory among patients with osteoarthritis undergoing total hip replacement surgery. Health Qual Life Outcomes.

[58] Li KK, Harris K, Hadi S, Chow E (2007). What should be the optimal cut points for mild, moderate, and severe pain?. J Palliat Med.

[59] Souza LA, Pereira LV, de Moura LA, Díaz LJ, da Cruz DD, Aparecido Da Silva J (2021). Structural validity of the chronic pain coping inventory-Brazilian version. PLoS One.

[60] Romano JM, Jensen MP, Turner JA (2003). The Chronic Pain Coping Inventory-42: reliability and validity. Pain.

[61] Jensen MP, Turner JA, Romano JM, Lawler BK (1994). Relationship of pain-specific beliefs to chronic pain adjustment. Pain.

[62] Lam ET, Lam CL, Fong DY, Huang WW (2013). Is the SF-12 version 2 Health Survey a valid and equivalent substitute for the SF-36 version 2 health survey for the Chinese?. J Eval Clin Pract.

[63] Damásio BF, Andrade TF, Koller SH (2015). Psychometric Properties of the Brazilian 12-Item Short-Form Health Survey Version 2 (SF-12v2). Paidéia (Ribeirão Preto).

[64] Kim SH, Jo MW, Ahn J, Ock M, Shin S, Park J (2014). Assessment of psychometric properties of the Korean SF-12 v2 in the general population. BMC Public Health.

[65] Młyńczak K, Golicki D (2022). Psychometric properties of the Polish version of SF-12v2 in the general population survey. Expert Rev Pharmacoecon Outcomes Res.

[66] Hayes CJ, Bhandari NR, Kathe N, Payakachat N (2017). Reliability and validity of the Medical Outcomes Study Short Form-12 Version 2 (SF-12v2) in adults with non-cancer pain. Healthcare (Basel).

[67] Bhandari NR, Kathe N, Hayes C, Payakachat N (2018). Reliability and validity of SF-12v2 among adults with self-reported cancer. Res Social Adm Pharm.

[68] Shah RM, Banahan BF, Holmes ER, Patel AS, Barnard M, Khanna R, Bentley JP (2018). An evaluation of the psychometric properties of the sf-12v2 health survey among adults with hemophilia. Health Qual Life Outcomes.

[69] Huo T, Guo Y, Shenkman E, Muller K (2018). Assessing the reliability of the short form 12 (SF-12) health survey in adults with mental health conditions: a report from the wellness incentive and navigation (WIN) study. Health Qual Life Outcomes.

[70] Rutter LA, Brown TA (2017). Psychometric properties of the Generalized Anxiety Disorder Scale-7 (GAD-7) in outpatients with anxiety and mood disorders. J Psychopathol Behav Assess.

[71] Johnson SU, Ulvenes PG, Øktedalen T, Hoffart A (2019). Psychometric properties of the general anxiety disorder 7-item (GAD-7) scale in a heterogeneous psychiatric sample. Front Psychol.

[72] Spitzer RL, Kroenke K, Williams JB, Löwe B (2006). A brief measure for assessing generalized anxiety disorder: the GAD-7. Arch Intern Med.

[73] Dhira TA, Rahman MA, Sarker AR, Mehareen J (2021). Validity and reliability of the Generalized Anxiety Disorder-7 (GAD-7) among university students of Bangladesh. PLoS One.

[74] Pressler SJ, Subramanian U, Perkins SM, Gradus-Pizlo I, Kareken D, Kim J, Ding Y, Sauvé MJ, Sloan R (2011). Measuring depressive symptoms in heart failure: validity and reliability of the patient health questionnaire-8. Am J Crit Care.

[75] McGuire LC, Strine TW, Allen RS, Anderson LA, Mokdad AH (2009). The Patient Health Questionnaire 8: current depressive symptoms among U.S. older adults, 2006 Behavioral Risk Factor Surveillance System. Am J Geriatr Psychiatry.

[76] Kroenke K, Strine TW, Spitzer RL, Williams JB, Berry JT, Mokdad AH (2009). The PHQ-8 as a measure of current depression in the general population. J Affect Disord.

[77] Ferguson L, Scheman J (2009). Patient global impression of change scores within the context of a chronic pain rehabilitation program. J Pain.

[78] Bobos P, MacDermid J, Nazari G, Furtado R, CATWAD (2019). Psychometric properties of the global rating of change scales in patients with neck disorders: a systematic review with meta-analysis and meta-regression. BMJ Open.

[79] Eremenco S, Chen WH, Blum SI, Bush EN, Bushnell DM, DeBusk K, Gater A, Nelsen L, Coons SJ, PRO Consortium’s Communication Sub committee (2022). Comparing patient global impression of severity and patient global impression of change to evaluate test-retest reliability of depression, non-small cell lung cancer, and asthma measures. Qual Life Res.

[80] Rampakakis E, Ste-Marie PA, Sampalis JS, Karellis A, Shir Y, Fitzcharles M (2015). Real-life assessment of the validity of patient global impression of change in fibromyalgia. RMD Open.

[81] Dworkin RH, Turk DC, Wyrwich KW, Beaton D, Cleeland CS, Farrar JT, Haythornthwaite JA, Jensen MP, Kerns RD, Ader DN, Brandenburg N, Burke LB, Cella D, Chandler J, Cowan P, Dimitrova R, Dionne R, Hertz S, Jadad AR, Katz NP, Kehlet H, Kramer LD, Manning DC, McCormick C, McDermott MP, McQuay HJ, Patel S, Porter L, Quessy S, Rappaport BA, Rauschkolb C, Revicki DA, Rothman M, Schmader KE, Stacey BR, Stauffer JW, von Stein T, White RE, Witter J, Zavisic S (2008). Interpreting the clinical importance of treatment outcomes in chronic pain clinical trials: IMMPACT recommendations. J Pain.

[82] Saldaña J (2013). The Coding Manual for Qualitative Researchers. 2nd edition.

[83] Cornish P, Churchill A, MacKay T (2020). Wellness together Canada: psychologists leading Canada's COVID-19 mental health response. Psynopsis: Canada's Psychology Newspaper.

[84] Stinson JN, Birnie KA, Noel ME, Ahola Kohut SH, Baerg K, Barwick M, Battaglia M, Boerner K, Campbell F, Carreiro E, Cornish P, Dick B, Dore-Bergeron M, Findlay S, Finley G, Gill J, Hudspith M, Ingelmo P, Killackey T, Lalloo C, Lamontagne C, Mohabir V, Nishat F, Oberlander T, Palermo T, Pham Q, Poolacheria Y, Poulin P, Rash J, Rasic N, Soltani S Evaluating a virtual stepped care portal in youth awaiting tertiary chronic pain care: an implementation-effectiveness hybrid type III study. Canadian Institutes of Health Research.

[85] Devan H, Hale L, Hempel D, Saipe B, Perry MA (2018). What works and does not work in a self-management intervention for people with chronic pain? Qualitative systematic review and meta-synthesis. Phys Ther.

[86] Wilson RA, Cooper L, Bosma R, Poulin P, Radhakrishnan A, Shergill Y, Bisson E, DiRenna T, Lalloo C, Mahar A, McEwen V, Olson J, Presseau J, Rash J, Snelgrove-Clarke E, Stinson J, Sud A, Telner D, van AZ, Vesnaver E, Visca R, Chandrasena C, Lall R, Motamedi F, Rhee K, Rifai T, Root-Clarke K, Skory L, Zur NN EmPOWERing primary care settings to use digital health interventions for chronic pain. Canadian Institutes of Health Research.

[87] Poulin PA, Lopatina E, Leroux JF, Wiebe P, Bosma R, Clarke H, Curci M, Di RT, Furlan A, Kittelberg L, Lomanowska A, Olson J, Peer M, Rash J, Shergill Y, Visca R, Ward S Improving chronic pain care with and for indigenous peoples: planning for the co-development and implementation of indigenous chronic pain resources using a two-eyed seeing approach. Canadian Institutes of Health Research.

